# Contrast-Enhanced Ultrasound-Guided Microwave Ablation for Iatrogenic Hepatic Hemorrhage: A Feasibility Study on Precision Hemostasis

**DOI:** 10.3390/bioengineering12060584

**Published:** 2025-05-28

**Authors:** Qing Li, Yi Liu, Zenghui Han, Xuan Zhou, Jianwei Wang, Xiaodong Zhou, Li Yan

**Affiliations:** 1 Ultrasound Diagnosis & Treatment Center, Xi’an International Medical Center Hospital, Xi’an 710100, China; lqgjyx369@sina.com (Q.L.); 13689280860@163.com (Y.L.); bingomars@sina.com (Z.H.); zx35072271@163.com (X.Z.); 15686752444@163.com (J.W.); 2 Institute of Medical Research, Northwestern Polytechnical University, Xi’an 710072, China

**Keywords:** microwave ablation, contrast-enhanced ultrasound (CEUS), liver puncture bleeding, liver, hemostasis, diagnosis and treatment

## Abstract

**Objectives**: The aim of this study was to investigate the feasibility of contrast-enhanced ultrasound (CEUS)-guided microwave ablation for managing iatrogenic hepatic hemorrhage following percutaneous liver puncture. **Materials and methods**: This retrospective study analyzed six patients (5 males, 1 female; mean age 56.8 ± 12.3 years) with CEUS-confirmed active hepatic hemorrhage refractory to 10 min compression and Agkistrodon halflorum hemagglutinase administration after percutaneous liver puncture (2023–2024). Etiologies included portal vein cavernous transformation (n = 4) and therapeutic intervention complications (n = 2). All patients underwent CEUS-guided microwave ablation comprising three phases: bleeding site localization, real-time ultrasound-guided ablation, and immediate postprocedural verification (CEUS: n = 6; DSA: n = 2). The protocol was approved by the institutional ethics committee with written informed consent. **Results**: All six patients achieved immediate hemostasis (mean 2.8 min) through CEUS-guided microwave ablation with 100% technical/clinical success. Preprocedural localization combined color Doppler and CEUS, while intraoperative real-time guidance ensured precise microwave needle placement. Post-ablation verification relied on CEUS (n = 6) with DSA confirmation in two cases. No major complications occurred; one patient reported transient abdominal pain resolving spontaneously. All patients remained stable during 7-day follow-up with no delayed complications. **Conclusions**: This study suggests that CEUS-guided microwave ablation is a rapid, minimally invasive, and effective option for iatrogenic hepatic hemorrhage, warranting further validation in larger cohorts.

## 1. Introduction

With the increasing utilization of image-guided percutaneous hepatic interventions, cavernous transformation of the portal vein (CTPV)—which may result from congenital portal venous maldevelopment or acquired conditions such as cirrhosis—has emerged as a significant etiology of portal hypertension. Patients with CTPV frequently require transjugular intrahepatic portosystemic shunt (TIPS) placement, mandating preliminary percutaneous transhepatic portal vein access. Furthermore, the growing adoption of other percutaneous liver procedures, including tumor ablation and biliary drainage, has amplified clinical demands. Although the incidence of hepatic artery hemorrhage is low, severe artery injuries can lead to life-threatening hemorrhage [1,2]. Consequently, precise hemorrhage localization and minimally invasive hemostatic techniques have become pivotal in mitigating procedural risks and optimizing patient outcomes.

The inherent bleeding risk associated with this procedure stems not only from its invasive nature, but also from the frequent coexistence of liver cirrhosis and hypersplenism-induced thrombocytopenia in this patient population. Active bleeding caused by liver injury can be life-threatening in severe cases, and immediate hemostatic intervention is needed. This approach has become more common since the increase in the number of percutaneous minimally invasive interventional surgeries of the hepatobiliary system [3]. Traditional methods of hemostasis, such as clamping, suturing, compression, bleeding organ resection, and transcatheter vascular embolization [4], are limited by substantial trauma, long operative times, many complications, and the inability to respond quickly to acute bleeding. Therefore, in dealing with bleeding from iatrogenic hepatic hemorrhage caused by various clinical percutaneous liver puncture therapies, there is an urgent clinical need for a rapid, precise, effective, and safe method of hemostasis.

In recent years, microwave ablation has emerged as a safe and effective minimally invasive modality for tumor treatment, achieving tissue destruction through rapid localized heating-induced coagulation necrosis [5]. Characterized by operational simplicity, minimal trauma, and rapid recovery, this technique has demonstrated clinical efficacy across multiple tumor types including thyroid nodules [6], liver tumors [7], uterine fibroids [8] and breast tumors [9]. While widely adopted in oncology, its potential for managing iatrogenic vascular injuries remains insufficiently explored—particularly for hemorrhage control during percutaneous interventions. Notably, our research group has accumulated substantial preclinical experience in ultrasound-guided percutaneous microwave ablation integrated with CEUS, achieving reliable hemostasis in animal models of hepatic hemorrhage [3,10,11,12]. Building on this foundation, the present study aimed to preliminarily investigate the application of percutaneous microwave ablation in per-cutaneous liver puncture bleeding by using the existing sample size and the performance of CEUS in preoperative detection, intraoperative monitoring, and post-operative evaluation to provide patients with a safer and more effective treatment strategy.

## 2. Materials and Methods

### 2.1. Study Population

From January 2023 to December 2024, data of six patients aged 19–71 years who underwent ultrasound-guided percutaneous microwave ablation for bleeding after percutaneous liver puncture at Xi’an International Medical Center Hospital were retrospectively analyzed. Inclusion criteria required: ① CEUS-confirmed active hemorrhage from puncture tracts, ② hemodynamic stability (SBP > 90 mmHg), ③ failure of initial conservative measures (compression ≥ 30 min), and ④ ineligibility for emergent surgery or transcatheter embolization due to contrast allergy or vascular anatomy constraints. There were five male patients and one female patient. The average age was 56.83 years. All patients met the standard indications for undergoing percutaneous liver puncture. Post-procedure bleeding was observed in all cases, with varying degrees of severity. In five patients, minor bleeding occurred immediately following the procedure. In one patient, however, significant bleeding developed 4 h after the procedure, with coil dislodgement and subsequent hemoperitoneum. The study was approved by the Ethics Committee of Xi’an International Medical Center Hospital, and all patients provided signed informed consent.

### 2.2. Instruments and Materials

ACUSON Sequoia ultrasound equipment (Siemens, 5C1 convex array probe, Hitachi, Ltd., Tokyo, Japan), a Ruibo microwave therapy instrument, and a Z05 microwave needle (14 G, 20 cm in length with the microwave emission point located 5 mm from the tip, Nanjing Ruibo Medical Technology Co., Ltd., Nanjing, China) were utilized. The ultrasound contrast agent used was SonoVue (sulfur hexafluoride microbubbles with an average diameter of 2.5 μm, Bracco Suisse SA, Plan-les-Ouates, Switzerland). The local anesthetic was a lidocaine hydrochloride injection (specification: 5 mL: 0.1 g, Hunan Kelun Pharmaceutical Co., Ltd., Yueyang, China).

### 2.3. Treatment Methods

Preoperative preparations: The routine blood test, coagulation series, and liver function indicators of all patients at the time of liver puncture were checked to ensure that they met the indications for microwave ablation therapy. The treatment process, possible risks and precautions were explained to the patient in detail, and informed consent was obtained from the patients. Color Doppler ultrasound and CEUS were used to confirm the location, amount, and peripheral vascular distribution of liver bleeding. The dose of contrast agent was 2.4 mL, which was administered by bolus injection through the median cubital vein. The mechanical index was 0.10. Percutaneous microwave ablation was immediately prepared for hemostasis upon real-time CEUS detection of microbubble extravasation beyond the hepatic capsule.

### 2.4. Intraoperative Treatment

All procedures followed the appropriate surgical principles, and local anesthesia was administered [13]. After local anesthesia, the patient was laid in the supine position and routinely disinfected. The surgical drape was laid, the treatment site was reconfirmed and positioned by ultrasound. The needle placement scheme was planned. The skin was cut with a #11 knife, and a WGT-Z05 (14 G) microwave antenna (needle) was used; under ultrasound guidance, the microwave needle precisely punctured the hemorrhage point in the liver, the microwave therapeutic device was turned on with a power of 80 W, and the ablation duration was 2–3 min to ensure adequate coagulation zone (mean diameter: 2.5 cm) covering the bleeding focus. During the ablation procedure, real-time two-dimensional grayscale ultrasound imaging was employed to continuously monitor dynamic changes in the size of the ablation-induced vaporization zone. The vital signs and bleeding status of the patient were closely monitored, and the treatment parameters were adjusted in a timely manner. After the end of treatment (a complete microwave ablation cycle), the needle was withdrawn to the surface of the liver, and the needle was removed after heating the needle tract. CEUS was performed again to confirm that the bleeding site had stopped bleeding, with all procedures performed exclusively by the same chief physician (28 years of ablation experience).

For patients with a large bleeding volume, ultrasound-guided placement of the abdominal drainage tube after microwave ablation was performed. Under ultrasound guidance, an 18G-PTC needle was inserted into the left abdominal ascites, the guidewire was placed, the sheath was dilated. An 8-F Dior drainage tube was placed in the left abdominal ascites, and the drainage tube (20 cm built-in and 5 cm exposed) was left indwelling. The light red liquid was removed by draining. A drainage bag was connected externally to occlude the drainage tube.

### 2.5. Postoperative Management

After percutaneous microwave ablation for hemostasis, the microwave needle was removed, and the puncture site was compressed to stop bleeding. After the patient was observed for 30 min, color Doppler ultrasound and angiography were performed again (two patients were given additional digital subtraction angiography (DSA) examination) to confirm that there was no bleeding or complications around the puncture site on the liver surface.

### 2.6. Observation Indicators and Evaluation Criteria

The time to stop bleeding, the success rate of hemostasis, and the occurrence of complications were recorded. Successful hemostasis was defined as the complete cessation of bleeding after treatment, and no further hemostatic measures were needed. All patients underwent follow-up evaluations at 7 days’ post-procedure to assess treatment efficacy and late-onset complications. Complications included local pain, fever, and infection.

## 3. Results

### 3.1. Imaging Features

**Figure 1:**
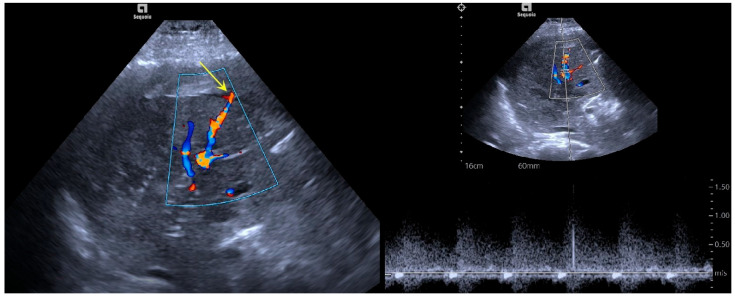
Color Doppler ultrasound manifestations before microwave ablation for hemostasis. The liver puncture needle track was filled with red and blue blood flow signals, some of which overflowed the liver capsule and took on a branch shape outside the liver capsule (indicated by the yellow arrow), while some of the blood flow signals went along the liver capsule. Spectral Doppler recorded high-speed blood flow spectra.

Preoperative color Doppler ultrasound suggested that the liver puncture needle track was full of red and blue blood flow signals, some of which overflowed the liver capsule and showed a branch shape outside the liver capsule, and some blood flow signals extended along the liver capsule. Spectral Doppler acquired a high-speed blood flow spectrum with a maximum speed of 100 cm/s (Figure 1).

**Figure 2:**
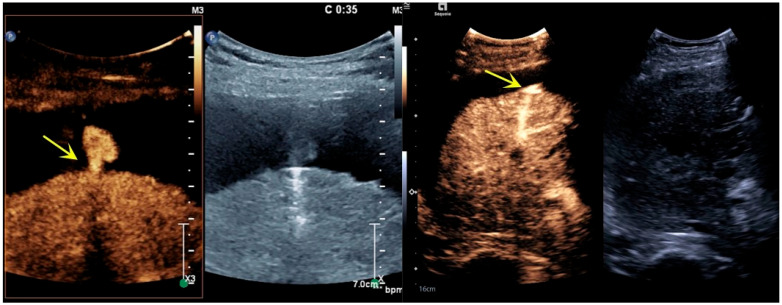
CEUS manifestations before microwave ablation for hemostasis. The contrast agent filled the liver puncture needle tract, and the signal was significantly stronger than that of the surrounding tissues. The contrast agent overflowed from the tear in the liver capsule (indicated by the yellow arrow), and some of the contrast agent was present in the prehepatic fluid dark area like a mushroom cloud.

**Figure 3:**
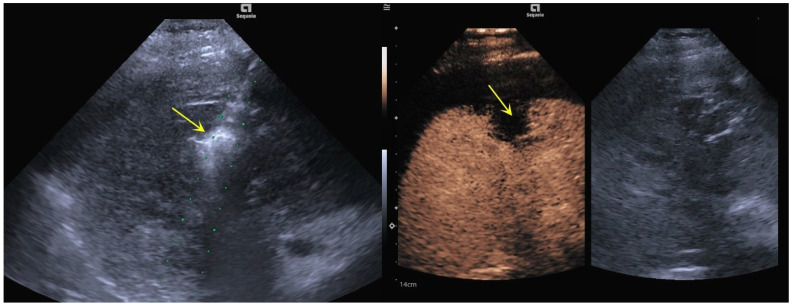
Two-dimensional ultrasound and CEUS manifestations after microwave ablation for hemostasis. Two-dimensional ultrasound suggested a strong echo of the ablation and gasification lesion in the liver puncture needle track. CEUS showed a contrast agent filling defect near the liver capsule in the liver puncture needle track. No contrast agent overflow along the liver capsule was observed (indicated by the yellow arrow). Guidance lines assist in visualizing the insertion path during the procedure (indicated by the green dots).

**Figure 4:**
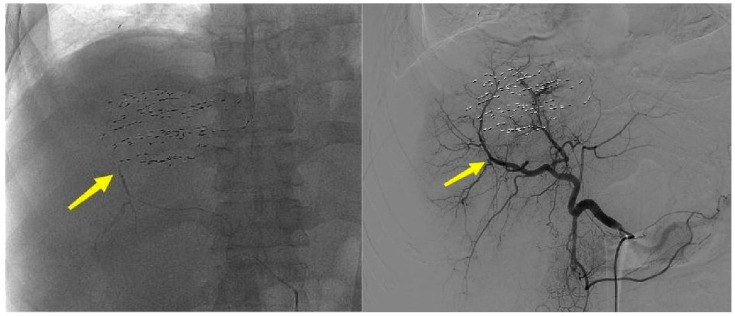
DSA manifestations after microwave ablation for hemostasis. Whole-liver angiography showed that the right posterior inferior branch was not developed, and the occlusion of the right posterior inferior branch of the liver was reconfirmed using an ultra-microcatheter (indicated by the yellow arrow).

Preoperative CEUS showed that the contrast agent filled the liver puncture needle tract, and the signal was significantly stronger than the signal of the surrounding tissues. The contrast agent overflowed from the liver capsule break, with a partial “mushroom cloud” appearance in the prehepatic fluid dark area (Figure 2). After microwave ablation for hemostasis, two-dimensional ultrasound showed strong echoes of ablative gasification foci in the hepatic puncture needle tract. Angiography showed a contrast agent filling defect near the liver capsule in the liver puncture needle track, and no contrast agent overflowed along the liver capsule (Figure 3). DSA images suggested that the interruption of the right posterior inferior branch of the hepatic artery was not visualized (Figure 4). Postoperative imaging data suggested that the liver puncture bleeding had disappeared.

### 3.2. Treatment Effect

The hemostasis of percutaneous microwave ablation was verified by imaging studies. Bleeding was successfully stopped in all six patients, showing a success rate of 100%. The average time for hemostasis was 2.8 min. No major damage to the liver or surrounding tissues was observed, with sustained hemostatic efficacy confirmed during 7-day follow-up evaluations. The patients who underwent microwave ablation for hemostasis did not experience significant complications during or after treatment. Only one patient had mild abdominal pain, but the symptoms disappeared after symptomatic treatment (Table 1). No delayed complications (e.g., rebleeding or infection) were detected in any case throughout the postoperative observation period.

## 4. Discussion

This study preliminarily confirmed that microwave ablation can quickly and effectively stop bleeding in patients with bleeding from liver puncture therapy. Only one patient had mild abdominal pain in the liver area. The main cause of the bleeding was low platelets. Platelets were lower than 10 × 10^9^/L before and after transjugular intrahepatic portosystemic shunt (TIPS) surgery. Although the puncture needle tract was initially embolized with coils, the patient’s severe thrombocytopenia (platelet count < 50 × 10^9^/L) led to partial coil migration beyond the hepatic capsule, resulting in active arterial hemorrhage (contrast extravasation on CEUS), which necessitated prolonged microwave ablation cycles to achieve hemostasis. The continuous delivery of 80 W for 3 min resulted in a larger ablation range. This patient had delayed bleeding, and the amount of blood loss was relatively large; a total of 3000 mL of nonclotting blood was drained from the abdominal cavity, which was the cause of this patient’s abdominal pain.

Active bleeding caused by liver injury can be life-threatening in severe cases, so immediate hemostatic intervention is needed. In patients with organ bleeding, surgical treatment is usually the first-line treatment used to stop the bleeding, but it has the disadvantages of substantial trauma, long operation time, many complications, and the risks brought by general anesthesia. In addition, patients with CPTV frequently present with concomitant liver cirrhosis and hypersplenism-induced thrombocytopenia. In such cases, surgical interventions carry a heightened risk of hemorrhage due to the invasiveness of extensive tissue dissection. Furthermore, embolotherapy necessitates prolonged preprocedural preparation and technically demands superselective catheterization, significantly extending procedural timelines. In recent years, with the development of minimally invasive treatment methods, the use of microwave ablation, as a means of ablation with minimal trauma, short time consumption, and accurate treatment, has gradually received more clinical attention. In microwave ablation, an emission antenna is inserted into the body to emit microwaves, which cause the rotation of water molecules and generate frictional heat, which is used to inactivate tumor tissues. Its advantages are that because it is less susceptible to the influence of the heat sink effect, the ablation range is easy to control, the ablation time is short, and it does not require the use of a counter plate [14]. Because of these advantages, microwave ablation has been used to control the bleeding of surface tumors, and the tumor achieved rapid hemostasis while the tumor shrunk [15]. In the treatment of liver cancer nodules [7], thyroid nodules [6], uterine fibroids [8], and breast tumors [9], rupture and bleeding can occur. Our team tested CEUS-guided percutaneous microwave coagulation therapy to control active hemorrhage in the rabbit liver and confirmed that microwave ablation can prevent bleeding in an active rabbit liver hemorrhage model. This study lays the foundation for animal experiments to support its application in humans with liver injury [10].

Ultrasound-guided percutaneous microwave ablation demonstrated an intrinsic capacity for rapid hemostasis through three synergistic mechanisms: ① real-time spatial localization of bleeding foci enabled by dynamic contrast-enhanced ultrasound monitoring; ② immediate induction of collagen denaturation and coagulative necrosis at the vessel-tissue interface via targeted microwave thermotherapy (80 W, 2–3 min); and ③ simultaneous obliteration of both the hemorrhage source and its perivascular microenvironment through controlled thermal diffusion. This integrated approach effectively bypasses the temporal limitations inherent to systemic pharmacological interventions [16,17] and avoids the angiographic navigation and contrast dependency of transarterial embolization (TAE) [18,19] while circumventing the extensive tissue trauma characteristic of open surgical hemostasis procedures [20,21].

In recent years, the new technology CEUS, consisting of a novel ultrasound contrast agent and angiographic imaging technology, has made major breakthroughs in the field of imaging medicine. This technique has made great contributions to medical research, clinical diagnosis, and treatment and has been hailed as a third-generation ultrasound revolution. The sulfur hexafluoride (SF_6_) microbubble contrast agent functions as a pure intravascular tracer—under physiological conditions, these microbubbles remain strictly confined within the vascular lumen, circulating with blood flow. Extravasation of microbubbles occurs exclusively at sites of vascular injury, where they escape through endothelial breaches, thereby enabling real-time ultrasonic detection of hemorrhage localization [22]. Efficient angiographic harmonic imaging technology and analysis technology are used to display and observe the blood flow perfusion status of the target in real time and perform quantitative analysis. Therefore, with regard to the efficacy of preoperative detection, intraoperative monitoring, and postoperative CEUS (S1), under ultrasound guidance, a percutaneous microwave needle can act precisely on the bleeding site and seal the bleeding blood vessels, minimizing damage to the patient’s surrounding tissues, shortening the time for hemostasis, and thus reducing bleeding. This is important for protecting liver function in patients and reducing the incidence of complications. Prolonged bleeding may lead to instability in the patient’s vital signs and increase the difficulty and risk of treatment. The rapid hemostatic ability of microwave ablation helps stabilize the patient’s condition and provides favorable conditions for later treatment [23].

Although this study has made some preliminary findings, it has several limitations. First, the small number of patients may have caused bias in the results. Because the incidence of hepatic artery hemorrhage is very low, despite the fact that we perform close to 2000 liver biopsy procedures annually, the need for multicenter trials is dire. The current study primarily focuses on emergency hemostasis. Future studies need to further explore its optimal operating parameters, indications, and long-term effects. Second, in real-world applications, doctors need to develop personalized treatment plans according to the specific conditions of each patient to ensure the safe and effective application of microwave ablation technology in the diagnosis and treatment of liver puncture bleeding. Third, the hemostatic mechanism and the optimization of the microwave ablation technique should be further investigated to provide stronger support for their use in clinical practice.

## 5. Conclusions

This study demonstrated that ultrasound-guided microwave ablation effectively controlled iatrogenic bleeding following percutaneous liver puncture. The technique achieved rapid hemostasis, providing a minimally invasive alternative to surgical intervention. While limited by sample size, the integration with CEUS enabled precise hemorrhage localization and treatment verification. These findings support further multicenter trials to optimize parameters and evaluate long-term outcomes.

## Figures and Tables

**Table 1:** Case data and the application of percutaneous microwave ablation on liver bleeding (TIPS: transjugular intrahepatic portosystemic shunt; CTPV: cavernous transformation of portal vein).

Case	Disease	Bleeding Location	Cause of Bleeding	Bleeding Time	Ablation Power/Time	Successful Hemostasis	Complications and Treatment
1	CTPV	Right anterior inferior branch	After percutaneous transhepatic portal vein puncture for TIPS Cirrhosis and Platelet deficiency	Immediately	80 W/120 s	Yes	None
2	Primary Liver Cancer	Right anterior inferior branch	Percutaneous puncture particle implantation	Immediately	80 W/120 s	Yes	None
3	CTPV	Right posterior inferior branch	After percutaneous transhepatic portal vein puncture for TIPS Cirrhosis and Platelet deficiency	Immediately	80 W/120 s	Yes	None
4	CTPV	Right posterior inferior branch	After percutaneous transhepatic portal vein puncture for TIPS Cirrhosis and Platelet deficiency	Immediately	80 W/120 s	Yes	None
5	CTPV	Right posterior inferior branch	After percutaneous transhepatic portal vein puncture for TIPS Cirrhosis and Platelet deficiency	4 h	80 W/180 s	Yes	Mild abdominal pain
6	Primary Liver Cancer	Right anterior inferior branch	Percutaneous puncture particle implantation	Immediately	80 W/120 s	Yes	None

## Data Availability

The original contributions presented in the study are included in the article, further inquiries can be directed to the corresponding authors.
